# MK2: an unrecognized regulator of tumor promoting macrophages in colorectal cancer?

**DOI:** 10.14800/macrophage.1166

**Published:** 2016-02-01

**Authors:** Eliseo F. Castillo, Anita L. Ray, Ellen J. Beswick

**Affiliations:** Department of Molecular Genetics and Microbiology, University of New Mexico Health Sciences Center, Albuquerque, NM 87131, USA

**Keywords:** MK2, inflammation, macrophages, colorectal cancer, colitis

## Abstract

Colorectal cancer (CRC) is one of the most common malignancies and is associated closely with inflammation before and after development. Macrophages promote colitis and colitis-associated CRC. M1 macrophages contribute to colitis directly through the production of proinflammatory cytokines and through activation of proinflammatory immune cell phenotypes. In cancer, both M1 and M2 macrophages participate in tumor development and progression through cytokine production, changes in cell signaling and activation of T cells. We have identified the mitogen-activated protein kinase-activated protein kinase 2 (MK2) as a regulator of macrophages during colitis-associated CRC (CAC). MK2 is a proinflammatory kinase that promotes production of IL-1α, IL-1β, IL-6 and TNF-α. MK2^−/−^ mice have decreases in macrophages, macrophage-associated chemokines, and proinflammatory cytokines. Most significantly, MK2^−/−^ mice do not develop neoplasms in an inflammatory model of CRC. However, addition of MK2^+/+^ macrophages to MK2^−/−^ mice increases production of proinflammatory cytokines. In wild type mice, both cytokines and tumor burdens increase upon addition of additional macrophages. These data support the importance of MK2 in macrophage regulation during inflammation-associated CRC.

CRC is one of the most common cancers in the US, affecting up to 1 in 5 people. In 2015, it is expected to kill nearly 50,000 people ^[1]^. Chronic inflammation is a risk factor for CRC, with risk correlating with severity and duration of colitis ^[2]^. Colitis and CAC involve many cell types and immune responses. Macrophages are key players in maintaining inflammation, both directly and indirectly by stimulating proinflammatory phenotypes in other cells. Here, we present an overview of the role of macrophages in CAC, as well as new evidence that MK2 is a regulator of macrophage function.

In response to colon inflammation, non-hematopoietic cells produce GM-CSF. For macrophages, GM-CSF stimulates polarization to the M1 phenotype and results in the production of high levels of IL-1β, IL-6 and TNF-α, which are characteristic M1 proinflammatory cytokines ^[3, 4]^. In early colitis, production of GM-CSF may help resolve acute inflammation through activation of regulatory T cells (Tregs). However, in cases of chronic inflammation, M1 macrophages continue to be a major source of cytokines to further drive inflammation ^[3]^.

IL-1β, IL-6 and TNF-α are all implicated in increasing the severity and duration of colitis in both mouse models and human patients ^[5]^. These cytokines change the endothelial environment to encourage recruitment of monocytes and activated T cells. Both IL-1β and IL-6 direct the differentiation of Th17 cells, which further promote inflammation through the production of cytokines. Th17 cells are also associated with development of colitis-associated cancers ^[6–9]^. Additionally, IL-6 promotes survival during inflammation, allowing epithelial cells to circumvent pro-apoptotic pathways to progress to colitis-associated cancer ^[10]^. TNF-α has many proinflammatory activities, but its ability to activate macrophages and T cells and prevent apoptosis in T cells are two of the main mechanisms for driving ongoing inflammation. Consequently, anti-TNF therapy has been used in human colitis with good effects for many patients, indicating that its multiple activities contribute strongly to chronic colitis ^[11]^.

M1 macrophages produce IL-12 to promote Th1 responses, and present antigen to preferentially induce Th1 or Th17 phenotypes ^[12]^. Th17 cells infiltrate during colitis in large numbers to produce IL-17, TNF-α and other proinflammatory cytokines. IL-17 can induce many other cells such as macrophages, fibroblasts and epithelial cells to produce IL-1β, IL-6 and TNF-α ^[13]^. Th17 cells promote Th1 activation and may also become Th1-like ^[14]^. Moreover, Th1 cells produce large amounts of TNF-α and are classically associated with colitis. Thus, it is no surprise that IL-17 blockade reduces the severity of colitis ^[15]^.

M2 macrophages have been found to resolve colitis. IL-10 is characteristically produced by M2 macrophages. IL-10 modulates the severity of colitis, and IL-10^−/−^ mice spontaneously develop disease. The major source of ameliorating IL-10 in colitis is from macrophages ^[16]^. However, as colitis progresses toward cancer, M2 macrophages begin to contribute to pathology.

As tumors develop in a CAC, the proportion of M1 macrophages decreases, while the proportion of M2 macrophages increases ^[17]^. M2 cells produce WNT ligands to activate the WNT signaling pathway in epithelial cells, which promotes survival and proliferation and reduces differentiation ^[18]^. As tumors progress, the prevalence of M2 macrophages increases and correlates with metastasis and chemoresistance ^[19, 20]^.

Macrophage trafficking differs depending on phenotype, allowing for regulated attraction of differentiated macrophages ^[21]^. Conditioned media from cancer cells increases migration of M2 cells, and the tumor microenvironment encourages M2 differentiation through IL-10 secretion ^[22, 23]^. This interplay between the tumor and macrophages supports an environment for tumor progression.

In many cancers, M1 macrophages are thought to fight cancer at an early stage of development, but in CRC the evidence is not as clear cut. Increases in M1 macrophages in some cancers decreases the risk for metastasis, but this is not the case in CRC ^[20]^. In part, the proinflammatory cytokines produced by M1 macrophages may drive pro-tumor activities. IL-1β helps primary tumors to invade tissue and maintains populations of stem-like cells ^[24]^. In mouse models, IL-6 levels do not decrease during the transition from colitis to tumor development, but instead increase as dysplasia progresses to tumors ^[25, 26]^. IL-6 also supports glycolysis, loss of differentiation, tumor growth and angiogenesis ^[27]^. In addition to cytokine production, one reason for this may be the induction of Th17 cells, which are associated with CRC development and progression ^[28]^. From initiation to metastasis, the cytokines produced by M1 macrophages (IL-1β, IL-6, and TNF-α) support CRC growth.

We recently examined these cytokines and the role of the MK2 pathway in CRC development by utilizing a mouse model of CAC ^[29]^. The generation of CAC was induced by AOM and several DSS treatments to simulate chronic colitis, resulting in colitis-associated neoplasm development in wild-type (WT) mice. Mice deficient for the MK2 gene were highly resistant to CAC development. Additionally, MK2-deficiency resulted in reduced IL-1α, IL-1β, IL-6 and TNF-α production as well as fewer macrophages in the colon under homeostatic and inflammatory states ^[29]^. Moreover, MK2^−/−^ macrophages produced far less anti-inflammatory IL-10 and also decreased proinflammatory cytokines IL-1α, IL-1β, IL-6 and TNF-α. These cytokines have all been implicated in regulating intestinal inflammation and CAC development ^[6, 30]^. Additionally, MK2^−/−^ mice are highly resistant to experimental colitis due to reduced IL-6 and TNF-α production, while IL-10 is later critical for taming colitis ^[31]^. Thus, the dampened inflammatory response in MK2^−/−^ mice is expected given the production of IL-1α, IL-1β, IL-6 and TNF-α are MK2-dependent ^[32–34]^. The reduction of colonic macrophages in MK2^−/−^ mice (steady-state and during inflammation) as well as their reduced cytokine production highlights a possible unrecognized role for the MK2 pathway in intestinal macrophage biology.

The genetic ablation of MK2 in mice drastically affects macrophage survival, accumulation/trafficking and function in the gut ^[29]^. The reduction of macrophages in the colon of MK2^−/−^ mice was quite surprising especially in unperturbed mice. Intestinal macrophages are the largest subpopulation of colonic mononuclear phagocytes and are considered a major population maintaining intestinal homeostasis ^[35]^. Intestinal macrophages are frequently replenished by monocytes as a normal part of intestinal homeostasis and during inflammation ^[36]^. Under homeostatic conditions, these monocytes give rise to intestinal macrophages that display an anti-inflammatory phenotype by producing IL-10 and exhibiting reduced responsiveness to toll-like receptor (TLR) signaling. During inflammation, these monocytes give rise to proinflammatory macrophages that are highly responsive to pathogen-associated molecular patterns ^[36–38]^. Nevertheless, it is unclear if MK2-deficiency resulted in rapid turnover and/or a decrease in maintenance of intestinal macrophages in the CAC model. However, we have generated bone marrow-derived macrophages (BMM) that differentiate and grow at similar rate to WT BMM (unpublished results). Thus, the decrease in macrophage numbers in the colon of MK2^−/−^ mice was unlikely due to macrophage differentiation.

Interestingly, we showed decreased levels of colonic GM-CSF and MCP-1 from AOM/DSS treated MK2^−/−^ mice compared to WT mice ^[29]^. GM-CSF can differentiate monocytes into M1 macrophages and is also important in macrophage activation, function, migration and survival ^[3,4]^. The chemokine, MCP-1, is involved in monocyte and macrophage migration to the gut ^[39]^. Additionally, MCP-1 plays an integral role in macrophage polarization by influencing expression of proinflammatory cytokines ^[40]^. Recent reports have shown MCP-1 is directly involved in macrophage migration to inflammatory sites in a MK2-dependent manner and that MCP-1 can directly activate MK2 ^[41]^. The role of MK2 in macrophage survival and trafficking appears to be unique to intestine since there is no difference in infiltrating macrophage numbers in MK2^−/−^ mice after spinal cord injury compared to control mice ^[42]^. Therefore, it is likely the selective reduction of intestinal macrophages in MK2^−/−^ mice is a phenotype that may be specific to the gut.

As previously mentioned, macrophages are abundant in the colon and are vital to maintain intestinal homeostasis ^[35]^. In the gut, macrophages shape T helper and Treg responses. These tissue specific macrophages are imprinted with an anti-inflammatory gene signature, allowing them to produce robust amounts of IL-10 to assist in the maintenance of Tregs ^[36–38]^. This anti-inflammatory population of macrophages is crucial to withstand acute insults in the gut that may otherwise initiate a lethal inflammatory response ^[39]^. The resolution of inflammation is in part dependent on resident macrophages producing IL-10 to inhibit proinflammatory responses of other innate cells and adaptive immune cells via an IL-10-STAT3-SOCS3 axis ^[43]^. This pathway is critical for gut homeostasis since IL-10-deficient mice or a deficiency in downstream signaling proteins (STAT3 and SOCS3) results in spontaneous intestinal inflammation as well as increased tumor incidence (in the AOM/DSS model) ^[43, 44]^.

During intestinal inflammation, monocytes traffic to the gut and differentiate into macrophages with a proinflammatory gene signature. This homing is dependent on MCP-1 and CCR2 expression ^[36, 45]^. Recently, MCP-1 was also shown to enhance LPS-induced IL-10 production from macrophages ^[40]^. MCP-1 appears to coordinate two vital events involved in intestinal macrophage generation by directing monocytes to the gut and by imprinting newly differentiated macrophages into IL-10-producing cells. Aligning with these past reports, we found low levels of MCP-1 as well as very few macrophages present in the colon of MK2^−/−^ mice compared to WT mice during inflammation. These macrophages also produced significantly lower amounts of IL-10 and expressed less arginase-1 (a prototypic M2 marker associated with an anti-inflammatory phenotype) ^[29, 46]^. Although MCP-1 levels are low in the colon of MK2^−/−^ mice during inflammation, it is still present at similar levels during steady-state conditions suggesting that other factors may be affecting MK2-deficient macrophages. This is understandable since MK2 signaling is a multifaceted event involving both intrinsic (p38, MK2, HSP27, AKT signaling complex) and extrinsic (GM-CSF, MCP-1) factors that regulate survival and migration, respectively.

Nevertheless, the MK2 negative phenotype does not appear to pose a serious risk like IL-10^−/−^ mice since MK2^−/−^ macrophages (and mice) also lack the ability to produce MK2-regulated proinflammatory cytokines that contribute to spontaneous inflammation. On the other hand, when we adoptively transferred MK2-replete BMM into MK2^−/−^ mice during the AOM/DSS treatment, the levels of GM-CSF, MCP-1 and IL-6 detected in the colon were restored to WT levels ^[29]^. Although neoplasms did not develop in these mice, the inflammatory response was restored in the colon. Our data suggest that the MK2 pathway in a single population of cells can invoke a potent inflammatory response when transplanted to an inflammatory setting.

The balancing act between anti-inflammatory and proinflammatory macrophages during homeostatic and inflammatory states in the gut remains an enigma. Interestingly, MK2 may regulate both anti-inflammatory (IL-10, IL-6 and TNF-α) as well as proinflammatory (IL-1, IL-6, TNF-α) cytokines. Thus, MK2 may play a previously unrecognized role in driving an intermediate macrophage phenotype that has both M1 and M2 properties. The M1 and M2 phenotypes are plastic. M2 macrophages infiltrating tumors (and isolated peritoneal cells in vitro) can be converted to a proinflammatory M1-like phenotype ^[47]^. In addition, populations of macrophages with mixed phenotypes, particularly during disease, complicate the M1/M2 characterization ^[48]^. We observed plasticity in our experiments; although we cultured BMM in M-CSF, which encourages a M2 phenotype, the macrophages isolated from the colon after transfer were M1-like and produced proinflammatory cytokines ^[29]^. Recently, this dual role of MK2 in both anti-inflammatory and proinflammatory cell polarization has also been shown in dendritic cells ^[49]^. These data complicate our understanding of the role of macrophages in colitis and CRC. Nevertheless, it is clear MK2 can be linked to numerous factors involved in colitis and CAC development. MK2 can regulate MCP-1 which is involved in intestinal homing of macrophage precursors under steady-state and inflammatory conditions. MCP-1 can also activate MK2 as well as polarize macrophages to produce IL-10 upon TLR4 stimulation ^[45]^. Lastly, MK2 regulates TNF-α-induced SOCS3 expression. These components (IL-10, TNF-α, MCP-1 and SOCS3), are directly or indirectly regulated by MK2 and all play a vital role in macrophage governing and maintaining intestinal homeostasis. Thus, it is plausible to suggest that MK2 regulates macrophage polarization and inflammatory imprinting in the gut.

In our report, we investigated MK2 involvement in CAC and revealed MK2 is critical for CAC development. Additionally, we discovered that MK2 plays an important role in intestinal macrophage biology. Considering the critical role of MK2 in driving CAC development and that it has therapeutic potential, it is vital to further understand its role in macrophage biology.

## Abbreviations

CRC: colorectal cancer
MK2: mitogen-activated protein kinase-activated protein kinase 2
regulatory T cells: Tregs
CAC: colitis-associated cancer
WT: wild type
BMM: bone marrow-derived macrophages
AOM: azoxymethane
DSS: dextran sodium sulfate
TLR: Toll-like receptor
